# Early Regulatory and Th2-Associated Responses Shape Resistance to *Leishmania panamensis* Infection in C57BL/6 Mice

**DOI:** 10.3390/pathogens15050540

**Published:** 2026-05-17

**Authors:** Lizzi Herrera, Carlos M. Restrepo, Rodrigo Villalobos, Kissy Degracia, Jennifer Álvarez, Patricia L. Fernández

**Affiliations:** 1 Centro de Biología Celular y Molecular de Enfermedades, Instituto de Investigaciones Científicas y Servicios de Alta Tecnología (INDICASAT AIP), Ciudad del Saber, Panama City 7144, Panama; lmherrera@indicasat.org.pa (L.H.); crestrepo@indicasat.org.pa (C.M.R.); 2 Sistema Nacional de Investigación (SNI), Secretaría Nacional de Ciencia, Tecnología e Innovación (SENACYT), Ciudad del Saber, Panama City 7144, Panama; 3 Departamento de Patología, Hospital Santo Tomás, Panama City 7127, Panama; patologia@hst.gob.pa; 4 Escuela de Biotecnología, Facultad de Ciencias de la Salud Dr. William C. Gorgas, Universidad Latina de Panamá, Panama City 7155, Panama; kissymerieris_237@hotmail.com; 5 Escuela de Biología, Facultad de Ciencias Naturales, Exactas y Tecnología, Universidad de Panamá, Panama City 7156, Panama; jpena24@its.jnj.com

**Keywords:** *L. panamensis*, C57BL/6, BALB/c, Th1/Th2 paradigm, cytokines

## Abstract

Characterizing the specific interactions of *Leishmania* species with different host systems is essential for the development and validation of experimental infection models and for identifying potential therapeutic targets. *Leishmania* parasites elicit diverse host immune responses that result in different levels of disease severity. Here, we developed a murine model of *L. panamensis* infection and compared the responses of BALB/c and C57BL/6 mice following intradermal ear inoculation. BALB/c mice developed progressive ulcerative lesions associated with high parasite burden, whereas C57BL/6 mice exhibited a transient edema and maintained low parasite levels detected only at early stages of infection. C57BL/6 mice displayed early production of IL-13, IL-4, and IL-10, followed by delayed IFN-γ secretion. In contrast, BALB/c mice showed a mixed Th1/Th2 response at later stages of infection. Humoral responses also differed between strains, with BALB/c mice developing an early and sustained IgG1-dominated response, while C57BL/6 mice exhibited weak and delayed antibody production. These findings suggest that resistance to *L. panamensis* infection in C57BL/6 mice is associated with an early and transient Th2/regulatory response accompanied by a weak and delayed antibody production.

## 1. Introduction

Leishmaniasis is a sandfly-borne disease caused by the flagellate protozoa of the genus *Leishmania* spp. [1]. This disease is endemic in 99 countries around the world, affecting mainly tropical and subtropical regions [2,3,4]. Leishmaniasis presents a diverse set of clinical manifestations, including visceral leishmaniasis, which can be fatal if left untreated, and cutaneous and mucocutaneous leishmaniasis, which are responsible for high morbidity in endemic areas [5]. According to the WHO, leishmaniasis is re-emerging, uncontrolled, and has a worsening epidemiological situation with a growing incidence of infection [6].

Pathogenic *Leishmania* species are divided into two main subgenera, *Leishmania (Leishmania)* and *Leishmania (Viannia)*. Species belonging to the subgenus *L. (Viannia)* are found exclusively in Central and South America and mainly cause cutaneous leishmaniasis, although they can migrate to the nasopharyngeal area and cause the more severe mucocutaneous form [4,7,8]. *L. (Viannia) panamensis* is the species responsible for most cases of leishmaniasis in Panama [9]. Although the clinical manifestations of leishmaniasis are largely determined by the infecting *Leishmania* species, the host immune response is essential in dictating whether the infection is controlled or progresses.

Much of our understanding of the immunopathology of leishmaniasis comes from experimental studies using mice infected with *L. (Leishmania) major* [10,11,12]. These studies showed an apparent susceptibility to infection in BALB/c mice, when compared to the less susceptible C57BL/6 mice. The mechanisms involved in these differences in susceptibility to *L. major* infection highlighted the relevance of the Th1/Th2 cell balance. Whereas C57BL/6 mice control infection by the development of a Th1 protective immune response, the more susceptible BALB/c mice show a non-healing Th2-biased response characterized by the expression of interleukin-4 (IL-4), interleukin-10 (IL-10) and interleukin-13 (IL-13) [10,11,13].

Although the host genetic background strongly influences susceptibility to *Leishmania* infection, multiple studies have shown that the infecting parasite species [13,14] and even the specific strain [15] constitute the primary determinants of the type of response that is developed. The study of the immune mechanisms behind the response to *L. (Viannia)* species has been limited because these species exhibit low infectivity in mice and typically induce self-healing lesions [16,17]. Nevertheless, some models of *L. panamensis* infection in BALB/c mice have observed a mixed Th1/Th2 phenotype, similar to that observed in humans [16,18]. These studies have found production of IFN-γ, IL-4, IL-13, and IL-10 in restimulated draining lymph nodes from infected animals in both the ear and the hind footpad.

Although there is evidence of some of the mechanisms associated with susceptibility to *L. panamensis* infection, much less is known about those associated with resistance. Using a footpad model of *L. panamensis* infection, we previously showed that C57BL/6 mice were resistant, with less footpad inflammation and parasitic loads than BALB/c mice [19]. This resistant phenotype was accompanied by low levels of IL-4 and IL-10 in draining lymph nodes restimulated in vitro at later time points of infection, although high levels of IL-13 were also detected. Moreover, in vitro stimulation of C57BL/6 macrophages with this parasite strain induced a gene expression pattern consistent with an M1-like macrophage phenotype [20].

In this study, we show a resistant phenotype of C57BL/6 mice in comparison with BALB/c using a model of *L. panamensis* ear infection. The resistance observed in C57BL/6 mice was associated with markedly elevated IL-13 levels and a transient production of IL-4 by draining lymph node cells during the early stages of infection. In addition, these mice maintained very low serum concentrations of IgG1 and IgG2a immunoglobulins throughout the course of infection. Collectively, these findings suggest a potential role for a Th2 early response in mediating resistance to *L. panamensis* infection in C57BL/6 mice.

## 2. Materials and Methods

### 2.1. Animals

Eight-week-old female BALB/c and C57BL/6 mice were provided by INDICASAT AIP animal facility. The animals were maintained at a constant temperature of 24 °C under a 12-h light/dark cycle, and unrestricted access to food and water. The Institutional Animal Care and Use Committee of INDICASAT AIP (IACUC-14-002) approved all experimental procedures, which were performed under the rigorous observance of the ethical standards pertaining to the handling of laboratory animals in compliance with both INDICASAT AIP and international regulations.

### 2.2. Parasites and Infection

Promastigotes of *L. panamensis* from a local patient with cutaneous leishmaniasis, strain PSC-1 (MHOM/PA/94/PSCI-1), were cultured at 25 °C in Schneider’s medium (Sigma-Aldrich, St. Louis, MO, USA) supplemented with 20% fetal bovine serum (FBS) (Gibco, Waltham, MA, USA). Parasite virulence was maintained through periodic passages of stationary-phase promastigotes into the hind footpad of BALB/c mice. Parasites extracted from the lesion site were cultivated until they reached the stationary phase. BALB/c and C57BL/6 mice (*n* = 30 total; *n* = 5 per group) were intradermally (i.d.) injected in the ear with 1 × 10^5^ virulent *L. panamensis* stationary-phase promastigotes in 10 µL of phosphate-buffered saline (PBS) using a 30-gauge needle. Lesion development was evaluated weekly for a total of 12 weeks using a digital caliper (SE 784EC), measuring the diameter and thickness of the edema/lesion in the ear. At two-week intervals (2, 4, 6, 8, 10 and 12 weeks), five animals from each strain were euthanized for collection of submandibular lymph nodes and whole blood, as well as for determination of parasite burden at the inoculation site. Three independent experiments were conducted. Histopathological analysis was performed in a single group of five animals at 10 weeks post-infection.

### 2.3. Parasite Load Quantification

The two ear sheets were separated, and the dermal side was placed face down on a 70 μm nylon cell strainer (Falcon) and macerated [21]. The homogenate was washed with 1 mL of PBS. The parasite load was determined by limiting dilution analysis. Briefly, tissue homogenates were serially diluted in 96-well tissue culture plates (Corning, Glendale, AZ, USA) containing Schneider’s medium, supplemented with 20% of FBS. The parasite load was defined as the reciprocal of the highest dilution at which viable promastigotes were detected after 10 days of incubation.

### 2.4. Histopathology

BALB/c and C57BL/6 mice inoculated in the ear with 1 × 10^5^ promastigotes were euthanized at week 10 post-infection. Infected tissues were excised and fixed with 4% formaldehyde in PBS. Samples were then embedded in paraffin, cut into sections of 4 μm using a microtome, placed in a water bath at 45 °C, and then the deparaffinized sections were mounted onto a slide. Slides were dried and stained with hematoxylin–eosin (H&E) for histopathological analysis.

### 2.5. In Vitro Restimulation of Lymph Node Cells

Submandibular lymph nodes were recovered at 2, 4, 6, 8, 10 or 12 weeks post-infection (*n* = 5 per time point) and transferred to nylon cell strainers of 70 μm (Corning, Glendale, AZ, USA). Trypan blue (Merck, Darmstadt, Germany) exclusion was used to quantify live cells after cells were separated by mechanical disruption. Cells were plated at a density of 5 × 10^5^ cells per well in 48-well plates using 500 μL of RPMI supplemented with 10% FBS and 100 U/mL penicillin/streptomycin (Merck, Darmstadt, Germany). Cells were stimulated with 20 μg/mL of soluble *L. panamensis* antigen (SLA), prepared by ten freeze–thaw cycles followed by sonication in an ice bath. Cells were incubated for 72 h at 37 °C in 5% carbon dioxide (CO_2_). Supernatants were harvested for cytokine (IL-4, IL-10, IL-13 and IFN-γ) quantification.

### 2.6. Antibody Titers in Plasma

Production of IgG1 and IgG2a antibodies against *L. panamensis* was determined in the serum from BALB/c and C57BL/6 mice at 0, 2, 4, 6, 8, 10 or 12 weeks post-infection (*n* = 5 per time point). Serum samples were obtained from blood collected by intracardiac puncture. Antibody titers were determined by an in-house enzyme-linked immunosorbent assay (ELISA). Briefly, 96-well plates (Nunc Maxisorb™, Thermo Fisher Scientific, Waltham, MA, USA) were coated with 50 μg/mL of SLA, diluted in 0.1 M of carbonate–bicarbonate buffer (pH 9.0) and incubated overnight at 4 °C in a humid chamber. After washing, plates were blocked with PBS buffer containing 1% bovine serum albumin (BSA) (Merck, Darmstadt, Germany) for 2 h. Samples were serially diluted (1:2), starting at 1:200 and incubated for 2 h at 37 °C. Secondary rat anti-mouse antibodies were used at dilutions of 1:4000 for IgG1 and 1:1000 for IgG2a (Thermo Fisher Scientific, Waltham, MA, USA). The reaction was developed with SIGMAFAST™ o-phenylenediamine dihydrochloride (OPD) (Merck, Darmstadt, Germany).

### 2.7. Cytokine Measurements

Concentrations of IFN-γ, IL-4, IL-10, and IL-13 were measured by ELISA using DuoSet Kits (R&D System, Inc. Minneapolis, MN, USA) according to the manufacturer’s protocol. Plates were incubated with streptavidin conjugated to horseradish peroxidase (R&D Systems, Minneapolis, MN, USA), and tetramethyl benzidine (TMB) (Merck, Darmstadt, Germany) was used as substrate. Optical densities were read at 450 nm in a multi-detection microplate reader (Synergy HT-Biotek, Agilent, Santa Clara, CA, USA).

### 2.8. Statistical Analysis

Statistical analyses were performed using GraphPad Prism version 11.0.0 (GraphPad Inc., La Jolla, CA, USA). Differences between groups were evaluated by ordinary one-way ANOVA followed by Tukey’s multiple comparison post hoc test or the non-parametric Mann–Whitney test, as appropriate. A *p*-value < 0.05 was considered statistically significant. Data are presented as mean ± standard error of the mean (SEM) from two or three independent experiments.

## 3. Results

### 3.1. C57BL/6 and BALB/c Mice Exhibit Distinct Infection Phenotypes upon L. panamensis Challenge

**Figure 1:**
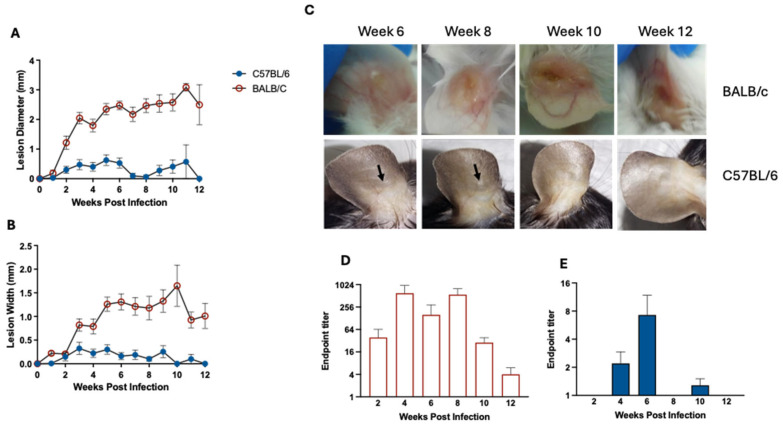
BALB/c mice develop larger lesions, and higher parasite loads than C57BL/6 mice following *L. panamensis* ear infection. BALB/c and C57BL/6 mice were inoculated in the ear with stationary-phase promastigotes (1 × 10^5^). Lesion development was evaluated weekly by measuring increases in ear diameter (**A**) and width (**B**). Results are presented as mean ± SEM from three independent experiments (*n* = 5 per group per experiment). (**C**) Representative photographs obtained from BALB/c and C57BL/6 ears at selected time points post-infection. Arrows indicate the transient edema in the site of inoculation. Parasite load in ears was determined by limiting dilution at 2, 4, 6, 8, 10 or 12 weeks post-infection in BALB/c (**D**) and C57BL/6 mice (**E**). End-point titers are represented as mean ± SEM from two or three independent experiments (*n* = 5 per group per experiment).

We first evaluated the progression of ear infection with *L. panamensis* in C57BL/6 and BALB/c mice. Animals were infected with 1 × 10^5^ stationary-phase promastigotes and lesion development was monitored for 12 weeks. We observed marked differences in lesion development between the two mouse strains (Figure 1A–C). As expected, BALB/c mice exhibited a constant increase in both ear lesion diameter and width, whereas C57BL/6 mice developed only a transient edema observed between 6 and 8 weeks post-infection that did not progress to ulcer formation. In contrast, BALB/c mice developed an initial edema that progressed to a papule by week 8 and finally evolved into an ulcer with a crater-like shape by week 10 post-infection (Figure 1C), a presentation that is characteristic of cutaneous leishmaniasis caused by *L. panamensis* in humans.

The parasite load was measured by limiting dilution throughout the course of infection. Parasites were detected at the inoculation site in BALB/c mice at all evaluated time points, with the highest burdens observed from week 4 through week 8 (Figure 1D). After week 8 of infection, the parasite load gradually declined, reaching very low levels by week 12. In contrast, C57BL/6 mice exhibited generally low parasite loads, peaking at week 6 post-infection (Figure 1E). After week 10, no parasites were detected, which is consistent with the evolution of the infection site in this mouse strain (Figure 1C,E). Together, these results indicate that C57BL/6 mice are less permissive to *L. panamensis* infection than BALB/c mice.

### 3.2. Differentially Organized Inflammatory Histopathological Patterns in BALB/c and C57BL/6 Mice

**Figure 2:**
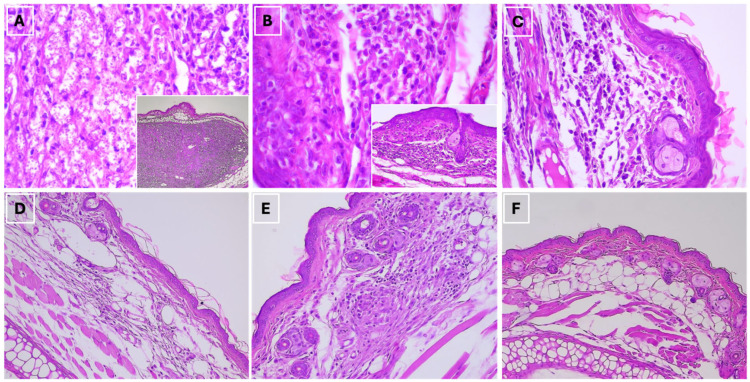
Histopathological analysis of *L. panamensis*-infected ear tissue in BALB/c and C57BL/6 mice showed the presence of inflammatory cell infiltrates at the inoculation site. Skin sections of BALB/c (**A**–**C**) and C57BL/6 (**D**,**E**) mice at 10 weeks post-infection were fixed and stained with hematoxylin and eosin. Images show 3 representative animals from each strain. Infected ear tissue section of BALB/c mice at 40× and 400× (**A**), 200× and 600× (**B**) and 400× (**C**), magnifications and infected C57BL/6 at 200× (**D**,**E**) and 100× (**F**) magnifications.

To characterize the histopathological differences to infection in the lesioned tissues of susceptible BALB/c mice and the self-resolving tissues of C57BL/6 resistant mice, histological examination of the infected ear pinnae was performed at 10 weeks post-infection. Both mouse strains showed inflammatory infiltrates (Figure 2). In BALB/c mice, we observed a marked tissue thickening and formation of granulomatous lesions, composed by macrophages, lymphocytes and some neutrophils (Figure 2A,B). These lesions displayed a disorganized architecture, and in some cases abundant amastigotes were detected within vacuolated macrophages (Figure 2A). Additionally, some animals exhibited a diffuse chronic inflammatory infiltrate (Figure 2C). No evidence of necrosis or vascular damage was observed in these samples. In contrast, C57BL/6 mice showed mild tissue thickening and a mild-to-moderate diffuse inflammatory infiltrate with a mixture of mononuclear cells (likely lymphocytes and macrophages) which were not arranged in an organized structure characteristic of a granulomatous pattern (Figure 2D–F). Amastigotes were not observed in the C57BL/6 samples.

### 3.3. BALB/c Mice Show a Mixed Th1/Th2 Immune Response to L. panamensis, Whereas C57BL/6 Mice Exhibit an Early Th2-Biased Immune Profile

**Figure 3:**
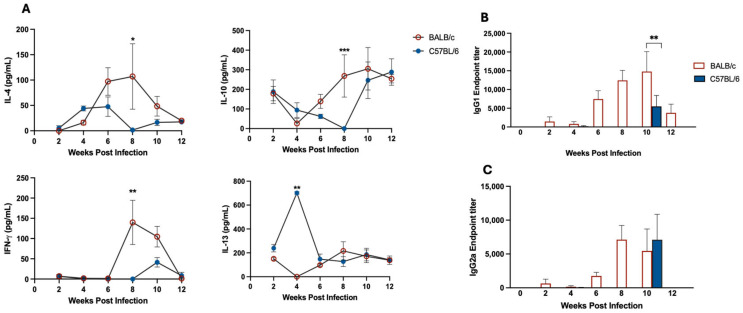
Distinct immune response patterns between BALB/c and C57BL/6 mice during *L. panamensis* infection. Submandibular lymph node cells from mice infected in the ear were collected and restimulated in vitro with soluble *L. panamensis* antigen (SLA). (**A**) Cytokine production after 72 h of SLA in vitro restimulation in cells from infected mice at 2, 4, 6, 8, 10, and 12 weeks post- infection. Results are represented as mean ± SEM from two or three independent experiments (*n* = 5 per group per experiment), each assayed in triplicate. *, *p* < 0.05, **, *p* < 0.01, *** *p* < 0.001. End-point titers of IgG1 (**B**) and IgG2a (**C**) measured in the serum of BALB/c and C57BL/6 mice at different time points post-infection. Results are represented as mean ± SEM from two independent experiments (*n* = 5 per group per experiment), each assayed in triplicate. **, *p* < 0.01.

In order to evaluate the response of both mouse strains to infection, levels of IFN-γ, IL-4, IL-10 and IL-13 were measured in the supernatant of lymph node cells restimulated with soluble *L. panamensis* antigen. BALB/c mice showed a mixed Th1/Th2 pattern with higher levels of IFN-γ, IL-4 and IL-10 than C57BL/6 mice at later stages of infection, reaching statistical significance at 8 weeks post-infection (Figure 3A). The production of IL-13 by BALB/c lymph node cells remained low throughout the infection period. Conversely, C57BL/6 cells produced higher levels of IL-13 as early as week 4 post-infection, reaching levels comparable to BALB/c by week 6 (Figure 3A). Surprisingly, although IL-10 production declined between weeks 4 and 8 post-infection, C57BL/6 mice exhibited elevated IL-10 levels, comparable to those of BALB/c mice at both the beginning and end of the experimental follow-up period. IL-4 levels in C57BL/6 mice were transiently elevated at weeks 4 and 6 (*p* = 0.0119 and *p* = 0.0173 compared to week 8, respectively). Unexpectedly, IFN-γ production in C57BL/6 mice remained low throughout the infection, with only a modest peak observed at week 10 (Figure 3A).

The antibody response to *L. panamensis* was also evaluated in the serum of infected animals. BALB/c mice showed a progressive increase in Th2- and Th1-associated antibody isotypes, IgG1 and IgG2a, respectively (Figure 3B,C). In this mouse strain, the IgG2a response occurs later than the IgG1 response, peaking at week 8 post-infection and subsequently stabilizing over time (Figure 3C), while IgG1 increases consistently starting from week 6 (Figure 3B). By week 12, levels of both antibody isotypes decreased or became undetectable. In the case of C57BL/6 mice, both antibody isotypes were detectable only at week 10 (Figure 3B,C), with IgG2a secretion appearing higher than that of IgG1.

## 4. Discussion

It has been established that resolution of infection caused by intracellular pathogens depends on the predominance of a Th1-type response, with IFN-γ as a central mediator, which has been associated with resistance. In contrast, susceptibility has classically been linked to a Th2 predominance associated with IL-4 production [12,22,23]. Infection with *Leishmania major* constitutes a classic example in which a Th1 response is required for parasite control, whereas a polarized Th2 response, characterized by elevated IL-4 and IL-13, is associated with parasite persistence [10,24,25]. However, accumulating evidence has highlighted that the immune response to *Leishmania* is far more complex than the Th1/Th2 dichotomy [24,26]. Moreover, there is robust evidence demonstrating that the response to *Leishmania* infection varies substantially across species and even among strains [13,24], influenced by evolutionary divergence between New and Old World species and by the subgenus to which each species belongs [13].

In the context of *L. panamensis* infection, chronicity appears to be associated with a mixed Th1/Th2 response phenotype in both humans and mice [16,18,27]. Nevertheless, the mechanisms underlying resistance to this *Leishmania* species have not yet been characterized. To address this gap, we infected C57BL/6 and BALB/c mice in the ear with *L. panamensis* and evaluated lesion development alongside the immune response of both mouse strains. Our preliminary results indicate that BALB/c mice are susceptible to *L. panamensis* infection, as evidenced by progressive lesion development, high parasite load, and the production of IFN-γ, IL-10, and IL-4 by draining lymph node cells. In contrast, C57BL/6 mice did not develop lesions and maintain a very low parasite burden, which is associated with an early transient production of IL-13, IL-4 and IL-10, followed by delayed and comparatively low levels of IFN-γ secretion.

For the infection, we used a strain of *L. panamensis* obtained from a local patient and adapted to mice through progressive passages in BALB/c over 24 months. Mice were inoculated with 1 × 10^5^ stationary-phase promastigotes and infection was monitored for a period of 12 weeks. Consistent with previous studies, BALB/c mice developed ulcerative lesions that became evident by week 10 post-infection. This progression was associated with an early and sustained increase in parasite load. Notably, despite the worsening of the lesion, the parasite load decreased between week 8 and 10 and reached very low titers at week 12. These results suggest that, although mice can contain replicating parasites, additional pathological mechanisms may be involved in tissue damage. The histopathological findings in BALB/c mice correlate with lesion severity at week 10 and are consistent with previous reports by Muñoz-Durango et al. in 2022 [18]. The detection of amastigotes in only a subset of animals is consistent with reduced parasite burdens at this stage of infection.

On the other hand, C57BL/6 mice did not develop detectable inflammation or lesions at the inoculation site. However, at week 10, the histopathological analysis revealed the persistence of a chronic inflammatory infiltrate predominantly composed of mononuclear cells, which was more pronounced in some animals. Furthermore, parasites were transiently detected between weeks 4 and 6 post-infection, although at substantially lower titers than those observed in BALB/c mice. Interestingly, we found high IL-13 production by lymph node cells in these animals during the early stages of infection. This was accompanied by a transient increase in IL-4 production between weeks 4 and 6. The significantly higher levels of IL-13 and IL-4 could be influencing the transient persistence of parasites at the inoculation site during these time points.

IL-13 and IL-4 have been associated with susceptibility to infection by several *Leishmania* species [28,29,30,31]. The roles of these two cytokines in *Leishmania* infection are closely linked, as both share the IL-4Rα chain. Conflicting findings regarding the role of IL-4 in *Leishmania* infection have been reported and are attributed, at least in part, to the compensatory effects of IL-13. However, IL-13-specific roles independent of IL-4 have also been demonstrated [32,33]. Experimental evidence shows that IL-13 overexpression drives susceptibility to *L. major* and contributes to susceptibility and chronicity in *L. mexicana* infection [29,34]. Nevertheless, protective roles for these two cytokines have also been suggested for infection with *Leishmania* and other intracellular pathogens [29,35,36]. For instance, IL-4 can synergize with IFN-γ to enhance macrophage activation and parasite killing in *L. major* infection [35]. Additionally, IL-13 has been linked to protection from *L. major* infection, as IL-4−/− BALB/c mice but not IL-4Rα−/− were able to control parasite dissemination [32,37]. In the case of *L. panamensis*, however, IL-13 has been associated with susceptibility to the parasite in a hind footpad model of infection [16], where IL-13 deficiency was associated with increased IFN-γ production. Consistent with these findings, IFN-γ production in C57BL/6 mice in our model was observed only at later stages of infection, when IL-13 and IL-4 levels had declined, whereas IFN-γ was absent during the early phase, when both cytokines were elevated.

Recent studies suggest that a Th2 environment at early stages of *L. amazonensis* infection may exert a protective effect by limiting the monocytic host cell reservoir, reducing IFN-γ-mediated recruitment of these cells, and enhancing IL-10 production at the site of inoculation [38]. Similarly, a limited Th1 response has been described during early *L. mexicana* infection, which is associated with a reduction in monocyte recruitment that appears to be dependent on IL-10 [39]. In that context, the initial limited Th1 response has been associated with chronicity and the development of non-healing lesions. In our model, C57BL/6 mice also exhibited elevated IL-10 levels during the early phase of infection; thus, the resistance of these animals to *L. panamensis* may occur through a mechanism involving a short limited Th2 and regulatory responses early in the infection. Further studies are required to characterize the microenvironment at the inoculation site that may favor a resolution of infection in this model. It is also important to consider that parasite strains or species, the site of infection (e.g., footpad, ear, or base of the tail), and the host background may contribute to the variability and conflicting results observed across studies.

We have previously demonstrated that infection with *L. panamensis* in the hind footpad of both BALB/c and C57BL/6 mice does not result in lesion development in either strain [19]. However, BALB/c mice exhibited significantly greater edema than C57BL/6 mice, which was associated with a higher parasite burden and increased IL-4 production by lymph node cells at week 8 post-infection. At this time point, IL-13 levels were similar in both strains [19]. In addition, in vitro infection of C57BL/6 macrophages with *L. panamensis* induced high IL-10 production [20]. Transcriptomic analysis of these cells revealed upregulation of arginase 1 (*arg1*) in response to infection. Together, these findings suggest a consistent pattern of Th2 and regulatory mediators contributing to the response of C57BL/6 mice to *L. panamensis*, both in vitro and in vivo.

We also assessed the production of IgG1 and IgG2a, immunoglobulin isotypes commonly associated with Th2- and Th1-biased responses, respectively. Although antibodies are generally considered to play a limited protective role in *Leishmania* infection, the profile of antibody isotypes can serve as an indicator of the underlying Th1/Th2 polarization. Previous studies indicate that antibodies may actively contribute to disease progression rather than protection. For instance, the presence of IgG1, but not IgG2a, has been associated with increased susceptibility in *L. mexicana* in vivo infection [40]. Mechanistically, it has been shown that antibodies can facilitate the entry of *L. mexicana* complex amastigotes into macrophages via Fcγ receptors, thereby promoting parasite survival and replication [41]. In addition, the formation of antibody–parasite immune complexes has been shown to induce IL-10 production by macrophages, further contributing to disease progression [42]. Consistent with this mechanism, it has been shown that disruption of either IL-10 or Fcγ receptor signaling leads to resolution of *L. mexicana* infection in C57BL/6 mice, with enhanced IFN-γ production and reduced disease severity [43], further supporting a model in which IgG-Fcγ receptor interactions drive chronic infection. While regulatory T cells have been identified as the main source of local IL-10, contributing to parasite persistence in *L. major* infection [44], IL-10 production by macrophages at the infection site also contributes to parasite survival [42].

We observed herein that BALB/c mice produced IgG1 and IgG2a early during infection, with levels peaking between weeks 8 and 10. IgG1 titers were consistently higher than those of IgG2a and remained detectable until week 12. These results are consistent with a mixed Th1/Th2 response profile in BALB/c mice infected with *L. panamensis* [18]. In contrast, C57BL/6 mice exhibited delayed antibody responses, with both IgG1 and IgG2a becoming detectable only at later stages of infection. Notably, IgG1 levels in C57BL/6 were significantly lower than those observed in BALB/c mice. Together, these findings suggest that B cells play a limited role in infection in C57BL/6 mice, which aligns with the resistant phenotype observed during *L. panamensis* infection. Given the role of antibodies in promoting Fcγ receptor-mediated IL-10 production, the reduced antibody response in C57BL/6 mice may limit this regulatory pathway at the site of infection. However, considering that lymph node cells from C57BL/6 mice produce elevated levels of IL-10 at later stages, additional mechanisms independent of antibody-mediated signaling are likely involved in shaping the regulatory environment in this strain. Further studies are necessary to characterize the cellular sources and regulatory networks responsible for antibody-independent IL-10 production, as well as their role in shaping the local immune microenvironment during infection.

Our findings suggest that resistance to *L. panamensis* infection in C57BL/6 mice extends beyond a classical Th1-dominated response and involves an early and transient Th2/regulatory profile that could limit the initial recruitment of monocytic host cells. This response is accompanied by weak and delayed antibody production, which might enhance resistance by reducing Fcγ receptor-mediated IL-10 induction. In contrast, BALB/c mice develop progressive disease characterized by higher parasite burden, sustained IgG1-dominated humoral responses, and a mixed Th1/Th2 profile. These results underscore the importance of early immune regulation in determining infection outcomes and provide insights into host–parasite interactions in *L. panamensis* infection.

## Data Availability

The original contributions presented in this study are included in the article. Further inquiries can be directed to the corresponding author.
